# Association between High-Risk Pregnancy History and High-Efficacy Postpartum Contraception Use in North Carolina

**DOI:** 10.1007/s10995-026-04297-6

**Published:** 2026-08-25

**Authors:** Maya E. Cox-Terry, Elizabeth D. Enright, Godwin Y. Dogbey, James M. Edwards

**Affiliations:** 1 East Carolina University Brody School of Medicine, 2100 Stantonsburg Rd Greenville, Greenville, USA; 2 Campbell University School of Osteopathic Medicine, Lillington, NC USA; 3 WakeMed Physician Practices Maternal Fetal Medicine, Raleigh, NC USA

**Keywords:** Postpartum, Contraception, High-risk pregnancy, LARC

## Abstract

**Objective:**

To evaluate factors associated with postpartum use of high-efficacy contraception among postpartum individuals in North Carolina and to assess whether a history of high-risk pregnancy was associated with increased use of WHO Tier 1 contraceptive methods.

**Study Design:**

North Carolina Pregnancy Risk Assessment Monitoring System (PRAMS) data from 2012 to 2020 were analyzed. The analytic sample included 6,993 postpartum respondents. A composite high-risk pregnancy history index was created using self-reported and birth certificate variables including preterm birth, low birth weight, obesity, hypertension, diabetes, depression, and inadequate prenatal care. Multivariable logistic regression was used to evaluate factors associated with postpartum use of WHO Tier 1 contraception.

**Results:**

A cumulative sample of 13,866 postpartum persons were selected and invited to complete the survey; 6,993 responded and were considered participants. History of high-risk pregnancy was associated with slightly increased odds of postpartum Tier 1 contraception use (aOR 1.18, 95% CI 1.00–1.39, *p* < 0.05), although the association was modest. Other significant factors associated with high-efficacy contraception use included multiparity (aOR = 2.00, 95% CI 1.70 to 2.36), planned conception (aOR = 0.72, 95% CI 0.62 to 0.85), living infant (aOR = 2.85, 95%CI 1.42 to 5.72), postpartum contraception counseling (aOR = 0.36, 95% CI 0.28 to 0.45), postpartum depression screening (aOR = 1.40, 95% CI 1.05 to 1.89), Black and Asian races (aOR = 0.78, 95% CI 0.64 to 0.95 and aOR = 0.36, 95% CI 0.23 to 0.59, respectively), a bachelor’s or master’s degree (aOR = 0.64, 95% CI 0.50 to 0.82 and aOR = 0.66, 95% CI 0.48 to 0.89, respectively), and age groups 30–34 and 35–39 year-olds (aOR = 1.31, 95% CI 1.08 to 1.59 and aOR = 1.50, 95% CI 1.19 to 1.89, respectively).

**Conclusion:**

In this North Carolina population, high-risk pregnancy history was only modestly associated with postpartum use of high-efficacy contraception. Other findings of this study suggest that high efficacy postpartum contraception use is influenced by demographic, reproductive, and healthcare interaction factors beyond pregnancy risk status alone. Interventions focused on equitable, patient-centered counseling and access to postpartum contraception may improve reproductive planning among patients with high-risk pregnancies who desire pregnancy spacing or prevention.

## Significance

Postpartum healthcare visit attendance alone did not predict the use of WHO Tier 1 contraception, rather the content of these visits impacted the uptake of high-efficacy contraception. Continued improvements are needed in counseling patients with high-risk pregnancies about the risks and benefits of future pregnancies, timing of subsequent pregnancies, and contraception use to aid in planning pregnancies if desired.

## Introduction

High-risk pregnancy is broadly defined as a pregnancy complicated by maternal medical, obstetric, behavioral, or social conditions associated with increased risk of adverse maternal, fetal, or neonatal outcomes. Common contributors include chronic hypertension, diabetes mellitus, obesity, previous preterm birth, low birth weight delivery, inadequate prenatal care, and mental health conditions, all of which are associated with increased risks of maternal morbidity, mortality, and recurrent pregnancy complications (Miller et al., 2022; Mvunta et al., 2019; Soltsman et al., 2021). Individuals with these conditions may benefit from pregnancy spacing and preconception optimization of chronic health conditions prior to subsequent pregnancies, particularly when future pregnancy is desired (Miller et al., 2022).

Postpartum contraception is an important component of reproductive healthcare because it allows individuals to achieve their reproductive goals, including pregnancy prevention, pregnancy spacing, or planning future pregnancies after optimization of health conditions. Short interpregnancy intervals have been associated with adverse outcomes including preterm birth, low birth weight, and small-for-gestational-age infants (Vricella et al., 2019). For patients with medical or obstetric conditions that increase pregnancy-related risks, access to effective contraception may provide additional opportunities to improve health prior to a subsequent pregnancy (Miller et al., 2022).

The World Health Organization (WHO) tiered-effectiveness framework categorizes contraceptive methods according to typical-use effectiveness. Tier 1 methods, the most effective reversible and permanent contraceptive options, include intrauterine devices (IUDs), contraceptive implants, tubal sterilization, and partner vasectomy (Stanback et al., 2015). Previous studies examining postpartum contraceptive use among patients with high-risk pregnancies have produced mixed findings. A study from Thailand reported higher utilization of Tier 1 contraception among patients with high-risk pregnancies, particularly permanent contraception (Jaruamornjit et al., 2022). In contrast, a United States-based cohort study found that although patients with high-risk pregnancies expressed greater interest in highly effective contraception, actual uptake did not differ significantly from those without high-risk pregnancies (French et al., 2016). These conflicting findings suggest that factors beyond pregnancy risk status may influence postpartum contraceptive decision-making.

North Carolina provides an important setting in which to examine postpartum contraceptive utilization. The state continues to experience substantial maternal health challenges, including high rates of chronic disease, pregnancy-related morbidity, and persistent racial disparities in maternal outcomes. Hypertensive disorders, cardiovascular disease, mental health conditions, and substance use disorders remain major contributors to pregnancy-related mortality, while Black women experience disproportionately higher rates of severe maternal morbidity and mortality compared with White women (North Carolina Maternal Mortality Review Committee, 2024). Additionally, North Carolina has implemented several policies intended to improve access to postpartum contraception, including Medicaid reimbursement for immediate postpartum long-acting reversible contraception (LARC) placement prior to hospital discharge. At the same time, federally mandated Medicaid sterilization consent requirements may create barriers to desired permanent contraception for some patients. Understanding how these healthcare and policy factors intersect with pregnancy risk status may help inform reproductive healthcare delivery within the state.

Discussions of postpartum contraception must also acknowledge the historical and contemporary context of reproductive autonomy. North Carolina’s history of forced sterilization through the state Eugenics Board disproportionately affected Black women, individuals living in poverty, and other marginalized populations. These experiences continue to shape concerns regarding reproductive coercion, healthcare mistrust, and inequities in contraceptive counseling (Dehlendorf et al. 2011a, b). Consequently, efforts to improve postpartum contraceptive care should be grounded in principles of reproductive justice, emphasizing informed consent, shared decision-making, and support for individual reproductive goals rather than increasing uptake of any specific contraceptive method.

Although previous studies have examined postpartum contraception among patients with high-risk pregnancies, many have been limited to single institutions, specific patient populations, or clinical settings. The North Carolina Pregnancy Risk Assessment Monitoring System (PRAMS) provides a population-based dataset that captures self-reported reproductive, behavioral, and healthcare experiences among postpartum individuals across the state, allowing evaluation of postpartum contraceptive use in a large and geographically diverse population.

The primary objective of this study was to evaluate whether a history of high-risk pregnancy was associated with postpartum use of WHO Tier 1 contraception among North Carolina PRAMS respondents. Secondary exploratory analyses examined demographic, reproductive, and healthcare-related factors associated with postpartum Tier 1 contraceptive use. We hypothesized that respondents with a history of high-risk pregnancy would have greater odds of using WHO Tier 1 contraception postpartum compared with respondents without such a history. We further explored associations between postpartum contraceptive use and maternal age, race, educational attainment, parity, pregnancy intention, insurance status, healthcare interactions, and experiences of interpersonal violence during pregnancy.

## Methods

### Study Design and Data Source

This study was a cross-sectional secondary analysis of data from the North Carolina Pregnancy Risk Assessment Monitoring System (PRAMS) collected between 2012 and 2020, excluding 2013 due to unavailable statewide data. PRAMS is a population-based surveillance system developed by the Centers for Disease Control and Prevention (CDC) in collaboration with participating state health departments to monitor maternal experiences and health behaviors before, during, and after pregnancy in the United States (Shulman et al., 2018). North Carolina participates in PRAMS and surveys approximately 1,700 postpartum individuals annually using a stratified random sampling design derived from state birth certificate records.

Disproportionate numbers of women with low-birth-weight infants are selected to participate in PRAMS to adequately weight the samples to account for the underrepresentation of this population. Exclusion criteria utilized by North Carolina PRAMS included out-of-state births to North Carolina residents, in-state births to non-residents, records missing the mother’s surname, higher-order multiple gestations involving four or more infants, and births to mothers aged 12 years or younger. Survey administration included mailed questionnaires with repeated follow-up mailings for nonresponse, followed by telephone interviews when necessary. PRAMS survey responses are self-reported and linked with de-identified birth certificate data.

The present analysis included only respondents who completed at least part of the PRAMS survey. Individuals sampled by PRAMS but lacking survey responses were excluded from analysis. The final analytic sample therefore consisted of postpartum respondents with both birth certificate data and survey data available.

### Study Population and Analytic Framework

The primary objective of this study was to evaluate factors associated with postpartum use of high-efficacy contraception among postpartum individuals in North Carolina. A secondary objective was to assess whether a history of high-risk pregnancy characteristics was associated with postpartum uptake of World Health Organization (WHO) Tier 1 contraceptive methods.

Importantly, the analytic sample was not limited exclusively to respondents with a history of high-risk pregnancy. Rather, all eligible PRAMS respondents were included, and high-risk pregnancy history was evaluated as an exposure variable within the larger cohort.

### Definition of High-Risk Pregnancy History

A composite “high-risk pregnancy history” index was created a priori using maternal health characteristics and adverse obstetric outcomes identified in prior literature as associated with increased maternal or neonatal morbidity (American College of Obstetricians and Gynecologists [ACOG], 2021; Centers for Disease Control and Prevention [CDC], 2022). Respondents were classified as having a history of high-risk pregnancy if they reported one or more of the following conditions or outcomes:

- Preterm delivery of the index pregnancy (< 37 weeks gestation).
- Previous preterm birth.
- Low birth weight delivery in the index pregnancy (< 2500 g).
- Previous low birth weight delivery.
- Pre-pregnancy or gestational diabetes.
- Hypertensive disorders before or during pregnancy.
- Pre-pregnancy obesity (body mass index ≥ 30 kg/m²).
- No prenatal care during the index pregnancy.
- Depression before or during pregnancy.

Variables were identified using linked birth certificate data and PRAMS survey responses.

All components of the index were weighted equally. Because this study pooled seven distinct PRAMS cohorts with year-specific sampling weights and stratification procedures, a weighted composite index was not constructed to avoid introducing disproportionate influence from any single survey year. The authors acknowledge that inclusion of depression within the composite index may differ from traditional clinical definitions of “high-risk pregnancy”; however, depression was retained due to its established association with adverse perinatal outcomes, impaired healthcare utilization, maternal morbidity, substance use disorders, and maternal mortality in pregnancy and the postpartum period (ACOG, 2023; Gavin et al., 2005; Jarde et al., 2016). The American College of Obstetricians and Gynecologists recognizes perinatal mental health conditions as significant contributors to maternal morbidity and mortality and recommends routine screening and treatment during pregnancy and postpartum care (ACOG, 2023). This consideration is particularly relevant in North Carolina, where suicide and drug overdose are among the leading causes of pregnancy-associated maternal death according to the North Carolina Maternal Mortality Review Committee (North Carolina Department of Health and Human Services, 2022). Given these public health concerns, depression was included as part of the broader high-risk pregnancy history construct. Nevertheless, this broader operational definition likely contributed to a higher observed prevalence of “high-risk pregnancy history” within the analytic sample compared with national estimates and should be interpreted accordingly.

### Outcome Variable

The primary outcome was postpartum use of a high-efficacy contraceptive method. A dichotomous variable was created to identify respondents reporting use of WHO Tier 1 contraceptive methods postpartum, including intrauterine devices (IUDs), contraceptive implants, tubal ligation, or partner vasectomy (World Health Organization, 2018).

### Covariates

Covariates were selected based on prior literature examining postpartum contraceptive utilization, maternal healthcare access, and reproductive decision-making. Variables included maternal age, race and ethnicity, marital status, educational attainment, parity, pregnancy intention, infant mortality, and postpartum insurance status categorized as public insurance (Medicaid or Children’s Health Insurance Program [CHIP]), private/other insurance, or uninsured.

Additional healthcare-related and psychosocial variables included interpersonal violence during pregnancy by a current or former partner, attendance at a postpartum healthcare visit, prenatal counseling regarding postpartum contraception, and postpartum counseling related to contraception, birth spacing, depression, and contraceptive prescription receipt.

Insurance status was included given known structural barriers affecting access to immediate postpartum long-acting reversible contraception (LARC) and sterilization, particularly among Medicaid beneficiaries. During portions of the study period, reimbursement policies and federal sterilization consent requirements may have limited access to immediate postpartum Tier 1 contraceptive methods for publicly insured patients in North Carolina (Moniz et al., 2017; Borrero et al., 2014).

### Statistical Analysis

Descriptive statistics and frequencies were calculated for all study variables among the full analytic cohort and among respondents reporting postpartum Tier 1 contraceptive use. Missing data were retained and reported descriptively. Missingness resulted from respondents leaving survey items unanswered or selecting “prefer not to answer” where applicable. Nonrespondents to the PRAMS survey were excluded entirely prior to analysis.

Complete-case analysis was used for regression modeling; respondents with missing data for variables included in a given model were excluded from that analysis only. The proportion of missing data varied by variable and is reported in the study tables.

Multivariable binary logistic regression was performed to examine factors associated with postpartum use of Tier 1 contraception. Adjusted odds ratios (aORs), 95% confidence intervals (CIs), and p-values were calculated. Variables were entered simultaneously into the regression model based on theoretical and literature-supported relevance rather than stepwise selection procedures.

To improve interpretability, all regression findings were reported as increased or decreased odds of Tier 1 contraceptive use. Statistical significance was defined as *p* < 0.05. All analyses were conducted using IBM SPSS Statistics version 28 (IBM Corp., Armonk, NY).

## Results

Between 2012 and 2020 (excluding 2013 due to unavailable statewide data), 13,866 postpartum individuals were selected to participate in the North Carolina PRAMS survey. Of these, 6,993 respondents completed at least part of the survey and were included in the analytic sample, corresponding to an overall response proportion of 50.4%.

Among all respondents, 68.7% (*n* = 4,802) met criteria for the composite high-risk pregnancy history index. Overall, 29.7% (*n* = 2,078) of respondents reported postpartum use of a World Health Organization (WHO) Tier 1 contraceptive method, including intrauterine devices, contraceptive implants, tubal ligation, or partner vasectomy. Among respondents reporting Tier 1 contraceptive use, 71.6% (*n* = 1,487) had a history of high-risk pregnancy.

Participant demographic, obstetric, and healthcare-related characteristics are summarized in Tables 1 and 2. Missing data varied across variables because some respondents did not answer specific survey questions or selected “prefer not to answer.” Only survey respondents were included in analyses; sampled nonrespondents were excluded prior to analysis.

**Table 1:** Factors used to categorize 2012–2020 North Carolina PRAMS participants as having a history of high-risk pregnancy

High-Risk Pregnancy Risk Factor	Number of participants (*n* = 6,993)	Number missing
Preterm delivery of index birth (< 37 weeks gestation)	1,789 (25.6%)	3
previous preterm delivery	774 (19.4%)	3,010
low birth weight delivery of index birth (< 2500 g)	2,397 (34.3%)	1
previous low birth weight delivery	838 (21%)	3,000
Diabetes before pregnancy	227 (3.3%)	57
Diabetes during pregnancy	489 (11%) & 273 (11.1%)*	2,552 & 4,536*
High blood pressure before pregnancy	464 (6.7%)	58
High blood pressure during pregnancy	808 (18.2%)	2,552
Depression before pregnancy	878 (12.7%)	82
Depression during pregnancy	585 (13.3%)	2,584
Obesity (BMI > 30)	1,821 (27.2%)	294
No prenatal care	59 (0.9%)	98

*The survey questions about diabetes were phrased slightly differently between the 2 phases of PRAMS surveys used in the study period. Thus, the individual frequencies of respondents with diabetes are listed separately by their respective survey phase utilized. *Reported as separate variables for the two PRAMS phases used (2012–2015 and 2016–2020)

**Table 2:** Specific WHO Tier 1 contraceptive methods used postpartum as reported by 2012–2020 North Carolina PRAMS participants who were categorized by history of high-risk pregnancy

Contraception Method	All participants (*n* = 6,993)	History of High-Risk Pregnancy (*n* = 4,802)	Low-risk pregnancy (*n* = 2,191)
IUD	946 (17.2%)	627 (16.7%)	319 (18.2%)
Implant	323 (5.9%)	236 (6.3%)	87 (5%)
Tubes Tied	631 (11.3%)	519 (13.6%)	112 (6.4%)
Vasectomy	196 (3.6%)	120 (3.2%)	76 (4.3%)

### Multivariable Logistic Regression

Results of the multivariable logistic regression analysis examining factors associated with postpartum Tier 1 contraceptive use are presented in Table 3.

**Table 3:** Demographic characteristics and health insurance status postpartum of 2012–2020 North Carolina PRAMS participants

Demographic Factor	Total number participants (*n* = 6,993)	Total number participants using Tier 1 Postpartum (*n* = 2,078)
Maternal Age		
< = 17	90 (1.3%)	28 (1.3%)
18–19	249 (3.6%)	60 (2.9%)
20–24	1,302 (18.6%)	350 (16.8%)
25–29	1,970 (28.2%)	542 (26.1%)
30–34	2,064 (29.5%)	652 (31.4%)
35–39	1,026 (14.7%)	345 (16.6%)
> = 40	292 (4.2%)	101 (4.9%)
Race		
White	3,969 (56.8%)	1,208 (58.2%)
Black	1,452 (20.8%)	427 (20.6%)
American Indian	80 (1.1%)	26 (1.3%)
Asian	294 (4.2%)	43 (2.1%)
Other Race	980 (14%)	312 (15%)
Mixed Race	215 (3.1%)	61 (2.9%)
Hispanic	1,233 (17.6%)	378 (18.2%)
Marital Status		
Single/Other	2,613 (37.4%)	803 (38.6%)
Married	4,377 (62.6%)	1,275 (61.4%)
Maternal Degree of Education		
< = 8th grade	295 (4.2%)	77 (3.7%)
9-12th grade, no diploma	695 (10%)	213 (10.3%)
High School Diploma/GED	1,394 (20%)	433 (20.8%)
Some College, No Degree	1,439 (20.6%)	475 (22.9%)
Associate’s Degree	688 (9.9%)	239 (11.5%)
Bachelor’s Degree	1,512 (21.7%)	389 (18.7%)
Master’s Degree	724 (10.4%)	193 (9.3%)
Doctoral/Professional Degree	230 (3.3%)	53 (2.6%)
Health Insurance		
Private/Other	3,835 (54.8%)	1,099 (52.9%)
Public (Medicaid or CHIP)	2,018 (28.9%)	627 (30.2%)
Uninsured	1,216 (17.4%)	390 (18.8%)

After adjustment for maternal demographic, obstetric, and healthcare-related variables, respondents with a history of high-risk pregnancy had significantly greater odds of reporting postpartum Tier 1 contraceptive use compared with respondents without a high-risk pregnancy history (adjusted odds ratio [aOR] 1.18, 95% confidence interval [CI] 1.00–1.39, *p* < 0.05).

Multiparous respondents demonstrated approximately twofold greater odds of Tier 1 contraceptive use compared with primiparous respondents after adjustment for covariates (aOR 2.01, 95% CI [1.70–2.36], *p* < 0.001). Respondents reporting actively attempting conception at the time of pregnancy had lower odds of postpartum Tier 1 contraceptive use compared with respondents who reported an unintended index pregnancy (aOR 0.72, 95% CI [0.62–0.85], *p* < 0.001) (Table 4).

**Table 4:** Healthcare provider counseling and other health and social factors reported by 2012–2020 North Carolina PRAMS participants

Health and Social Factors	Total number of all participants (*n* = 6,993)	Total number of participants using Tier 1 postpartum (*n* = 2,078)
Previous Live Birth (Multiparity)	3,926 (57.2%)	1,437 (70.2%)
Index Infant Living	6,681 (98.2%)	2,027 (99%)
Pregnancy Intention (Trying to get pregnant with index pregnancy)	3,786 (54.9%)	981 (47.7%)
Using Contraception Postpartum	5,432 (79.4%)	1,980 (96.2%)*
Abuse by partner during pregnancy	132 (1.9%)	33 (1.6%)
Abuse by ex-partner during pregnancy (2016–2020 data only)	52 (1.2%)	15 (1.2%)
Healthcare Provider Counseling		
Asked about plan to use birth control postpartum (prenatal visit)	3,768 (85.5%)	1,140 (87.9%)
Asked about depression (prenatal visit)	3,727 (84.6%)	1,110 (85.7%)
Participant attended postpartum visit	6,192 (90.4%)	1,928 (93.5%)
Discussed contraception options (postpartum)	3,631 (90%)	1,011 (82.5%)
Asked about depression (postpartum visit)	3,736 (92.5%)	1,146 (93.5%)
Prescribed birth control (postpartum)	1,904 (47.3%)	315 (25.9%)
Discussed pregnancy spacing	2,130 (52.9%)	575 (47.1%)
Inserted IUD or implant (postpartum)	874 (21.9%)	664 (54.5%)

Missing data were removed from analysis. Valid total number of participants and percentage of participants are reported. *The survey contained questions about current use of contraception and questions about current contraception method specifically. It is likely that participants who selected their contraception method, particularly tubal sterilization or vasectomy, either did not respond to or selected “no” for survey question about current contraception use, thus the total did not add up to 100%

Respondents whose infant was living at the time of survey completion demonstrated significantly greater odds of Tier 1 contraceptive use compared with respondents reporting infant loss (aOR 2.85, 95% CI [1.42–5.72], *p* = 0.003). Additionally, respondents who reported being screened by a healthcare provider for postpartum depression symptoms had increased odds of Tier 1 contraceptive use relative to respondents who were not screened (aOR 1.40, 95% CI [1.05–1.88], *p* = 0.023) (Table 5).

**Table 5:** Significant predictors of high-efficacy contraception use postpartum among 2012–2020 North Carolina PRAMS participants (Cox-Terry, et al., 2024)

Variable	Coefficient (B)	aOR	95% CI	*p*-value
History of high-risk pregnancy	0.167	1.18	[1.00 to 1.39]	0.046
Multiparity	0.696	2.01	[1.70 to 2.36]	< 0.001
Trying to get pregnant with recent pregnancy*	−0.324	0.72	[0.62 to 0.85]	< 0.001
Living infant	1.046	2.85	[1.42 to 5.72]	0.003
Postpartum contraception counseling*	−1.036	0.36	[0.28 to 0.45]	< 0.001
Postpartum visit inquiry about depression	0.340	1.40	[1.05 to 1.88]	0.023
Black race*	−0.248	0.78	[0.64 to 0.95]	0.014
Asian race*	−1.010	0.36	[0.23 to 0.59]	< 0.001
Bachelor’s degree*	−0.443	0.64	[0.50 to 0.82]	< 0.001
Master’s degree*	−0.422	0.66	[0.48 to 0.89]	0.007
30–34 years-old	0.268	1.31	[1.08 to 1.59]	0.007
35–39 years-old	0.408	1.50	[1.19 to 1.89]	< 0.001

*All aORs less than 1 were inverted together with their corresponding 95% CIs for consistency of reporting values greater than 1

In contrast, respondents identifying as Black or Asian had lower odds of postpartum Tier 1 contraceptive use compared with White respondents. Black respondents demonstrated 28% lower odds of Tier 1 contraceptive use (aOR 0.72, 95% CI [0.64–0.95], *p* = 0.014), while Asian respondents demonstrated substantially lower odds of use (aOR 0.36, 95% CI [0.23–0.59], *p* < 0.001).

Educational attainment was also associated with contraceptive use patterns. Respondents with bachelor’s (aOR 0.64, CI [0.50–0.82], *p* < 0.001) or master’s degrees (aOR 0.66, 95% CI [0.48–0.89], *p* = 0.007) had approximately 50% lower odds of Tier 1 contraceptive use compared with respondents with a high school diploma or GED.

Maternal age demonstrated variable associations with postpartum Tier 1 contraceptive uptake. Compared with respondents aged 25–29 years, respondents aged 30–34 years had modestly increased odds of Tier 1 contraceptive use (aOR 1.31, 95% CI [1.08–1.59], *p* = 0.007), while respondents aged 35–39 years demonstrated approximately 1.5-fold greater odds of use (aOR 1.50, 95% CI [1.19–1.89], *p* < 0.001).

## Discussion

In this population-based analysis of North Carolina PRAMS data, respondents with a history of high-risk pregnancy demonstrated modestly increased odds of postpartum World Health Organization (WHO) Tier 1 contraceptive use compared with respondents without a history of high-risk pregnancy. However, the magnitude of this association was relatively small after adjustment for demographic, obstetric, and healthcare-related variables, suggesting that high-risk pregnancy history alone may not strongly influence postpartum uptake of highly effective contraception.

Several factors demonstrated stronger associations with postpartum Tier 1 contraceptive use than high-risk pregnancy history itself. Multiparity, maternal age between 30 and 39 years, postpartum depression screening, and having a living infant were associated with increased odds of Tier 1 contraceptive use. In contrast, Black race, Asian race, higher educational attainment, and intended pregnancy were associated with lower odds of postpartum Tier 1 contraception use.

Importantly, postpartum visit attendance and prenatal contraceptive counseling were not independently associated with Tier 1 contraceptive uptake. These findings suggest that the quality, timing, content, and context of contraceptive counseling may be more influential than counseling occurrence alone. Because PRAMS does not assess counseling quality or method-specific counseling content, the present study cannot determine whether respondents received comprehensive counseling regarding long-acting reversible contraception (LARC), sterilization, or pregnancy spacing.

The observed prevalence of respondents meeting criteria for the composite high-risk pregnancy history index (68.7%) was substantially higher than national estimates of high-risk pregnancy prevalence, which are generally reported between 6% and 12% (Centers for Disease Control and Prevention [CDC], 2022). Several factors likely contributed to this discrepancy. First, North Carolina experiences disproportionately high rates of pre-pregnancy overweight and obesity. In 2020, approximately 56.8% of North Carolina births occurred among individuals with pre-pregnancy overweight or obesity, exceeding national estimates (North Carolina Department of Health and Human Services [NCDHHS], 2024; Driscoll & Gregory, 2020). Second, PRAMS intentionally oversamples births involving low-birth-weight infants to improve representation of high-risk populations (Shulman et al., 2018). Consistent with this sampling strategy, low birth weight deliveries were overrepresented within the analytic sample compared with statewide birth statistics. Finally, the study utilized a broad public health-oriented composite index incorporating medical, obstetric, and behavioral risk factors rather than a narrowly defined clinical designation of “high-risk pregnancy.” These methodological considerations likely increased the proportion of respondents classified as high risk and should be considered when interpreting generalizability.

The observed racial disparities in postpartum Tier 1 contraceptive use likely reflect a complex interaction of structural inequities, healthcare access barriers, provider counseling differences, and historical mistrust of reproductive healthcare systems. Prior literature demonstrates that contraceptive counseling and reproductive healthcare delivery are shaped by implicit bias, differential counseling practices, and historical reproductive coercion experienced disproportionately by racialized and marginalized communities (Dehlendorf et al. 2011a, b; Gomez et al. 2014). These findings should therefore not be interpreted as evidence that increased uptake of Tier 1 contraception is universally desired across populations. Rather, equitable reproductive healthcare requires ensuring that all patients have access to accurate information and access to all methods of contraception, as well as autonomous contraceptive decision-making consistent with their reproductive goals.

This consideration is particularly important in North Carolina given the state’s history of involuntary sterilization through the North Carolina Eugenics Board. Although immediate postpartum LARC placement and sterilization may reduce unintended short-interval pregnancies and improve maternal outcomes for some patients, efforts to expand access must remain grounded in reproductive justice principles and shared decision-making rather than prioritizing contraceptive uptake alone (Roberts, 1997; Gomez et al., 2014).

The association between postpartum depression screening and increased Tier 1 contraceptive use may reflect broader engagement in comprehensive postpartum care rather than direct counseling regarding contraceptive effects on mood. Providers who conduct postpartum depression screening may also be more likely to engage patients in discussions regarding future pregnancy planning, reproductive goals, and optimization of maternal mental health prior to subsequent pregnancies. However, because PRAMS does not capture the timing, quality, or content of counseling interactions, these findings should be interpreted cautiously.

The finding that postpartum contraceptive counseling was associated with lower odds of Tier 1 contraceptive use warrants further investigation. One possible explanation is that some respondents received immediate postpartum sterilization or inpatient LARC placement prior to the postpartum visit and therefore may not have required additional outpatient contraceptive counseling. Alternatively, differences in counseling quality, patient preferences, healthcare access, or counseling timing may have influenced this association. Future studies should distinguish between immediate postpartum and interval contraceptive initiation and should evaluate patient-centered counseling approaches in greater detail.

These findings have important implications for maternal health policy and clinical care in North Carolina. Rural counties and maternity care deserts within the state continue to experience significant barriers to obstetric and reproductive healthcare access. Expanding equitable access to postpartum contraception, integrating reproductive planning with mental health screening, and improving access to immediate postpartum contraceptive services may help reduce unintended short-interval pregnancies among individuals who desire pregnancy prevention. However, interventions should prioritize patient autonomy, culturally responsive counseling, and shared decision-making rather than focusing solely on increasing uptake of specific contraceptive methods.

Future North Carolina-based research should evaluate geographic disparities in postpartum contraceptive access, distinguish between LARC and permanent contraception utilization, and examine how counseling quality, reproductive coercion concerns, and healthcare trust influence contraceptive decision-making among postpartum individuals with medically complex pregnancies.

## Limitations

This study has several important limitations. First, the definition of high-risk pregnancy used in this analysis was based on a study-specific composite index derived from available PRAMS survey and birth certificate variables rather than a standardized clinical definition. Although the index incorporated multiple obstetric and medical risk factors associated with adverse maternal outcomes, some included variables—particularly depression and obesity—may not align with narrower obstetric definitions of high-risk pregnancy. This broader operationalization likely contributed to the relatively high proportion of respondents classified as high risk within the analytic sample.

Second, the study relied on secondary, self-reported survey data linked with birth certificate information. Self-reported data are subject to recall bias, inaccurate reporting, and response misclassification, particularly regarding healthcare counseling experiences and contraceptive use. Additionally, PRAMS survey items were not designed specifically for this study’s research question, limiting the ability to assess detailed clinical information, contraceptive timing, counseling quality, or patient preferences.

Third, non-response bias is possible because only respondents who completed the PRAMS survey were included in the analysis. Individuals who did not respond may differ systematically from respondents in ways that influence contraceptive use, healthcare access, or pregnancy risk characteristics. Similarly, the PRAMS surveys are only available in English and Spanish, thus limiting the responses of populations in NC who speak other languages, such as Haitian-Creole or Arabic.

Fourth, the cross-sectional design precludes determination of causality or temporal relationships between measured variables and postpartum contraceptive use. Logistic regression models estimate odds of association rather than direct risk and cannot determine whether observed factors caused differences in contraceptive uptake.

Finally, because this study examined North Carolina PRAMS data specifically, findings may not be fully generalizable to populations in other states with different demographic compositions, healthcare systems, Medicaid policies, or postpartum contraceptive access environments.

## Conclusions

Within this North Carolina PRAMS cohort, a history of high-risk pregnancy was associated with modestly increased odds of postpartum WHO Tier 1 contraceptive use; however, demographic, obstetric, and healthcare-related factors demonstrated stronger associations with contraceptive uptake than high-risk pregnancy history alone. Significant racial and educational disparities in Tier 1 contraceptive use were identified, underscoring the importance of addressing structural barriers, healthcare inequities, and differences in counseling experiences.

These findings support continued efforts to improve equitable access to postpartum contraceptive services throughout North Carolina, particularly in rural and underserved communities. Future interventions should emphasize patient-centered counseling, reproductive autonomy, and shared decision-making while integrating pregnancy planning discussions into broader maternal healthcare and mental health services. Additional research is needed to better understand how counseling quality, immediate postpartum contraception availability, and healthcare system factors influence postpartum contraceptive decision-making among individuals with medically complex pregnancies.

## Data Availability

NC Pregnancy Risk Assessment Monitoring System data is de-identified, publicly available data. PRAMS NC Website and Contact Information: https://schs.dph.ncdhhs.gov/units/stat/prams/; State Center for Health Statistics, Division of Public Health, North Carolina Department of Health and Human Services, 2422 Mail Service Center, Raleigh, NC 27699-2422, Phone: (984) 236-7416.

## Code Availability

Not applicable. Used SPSS IMB 28^th^ Edition.
